# Genome-Wide Analysis of the *MsRCI2* Gene Family in *Medicago sativa* and Functional Characterization of *MsRCI2B* in Salt Tolerance

**DOI:** 10.3390/ijms26094165

**Published:** 2025-04-27

**Authors:** Huiru Qiao, Depeng Zhang, Zizhao Zhang, Jing Meng, Pin He, Shichao Zhang, Yan Wang, Hua Cai, Yong Li

**Affiliations:** College of Life Science, Northeast Agricultural University, Harbin 150030, China

**Keywords:** *Medicago sativa* L., *MsRCI2B*, ion homeostasis, ROS scavenging, protein interaction

## Abstract

The Rare Cold-Inducible 2 (*RCI2*) gene encodes a conserved hydrophobic peptide that plays a crucial role in ion homeostasis, membrane stability, and responses to abiotic stress. In this study, six members of the *MsRCI2* gene family were identified in *Medicago sativa* L., all of which contain highly conserved PMP_3_ domains. Comparative collinearity analysis revealed syntenic relationships between *M. sativa* and *M. truncatula*, with each gene displaying distinct expression profiles under various stress conditions. Among them, *MsRCI2B* was significantly upregulated in response to salt stress. Alfalfa plants overexpressing *MsRCI2B* exhibited enhanced salt tolerance, as evidenced by increased antioxidant enzyme activities and reduced accumulation of malondialdehyde (MDA), hydrogen peroxide (H_2_O_2_), and superoxide anion (O_2_^−^) compared to wild-type plants. Furthermore, the transgenic lines maintained better Na⁺/K⁺ homeostasis under salt stress, reflected by a lower Na⁺/K⁺ ratio and significantly elevated expression of key ion transport genes, including *MsSOS1*, *MsAKT1*, and *MsNHX1*. To elucidate the molecular mechanisms underlying *MsRCI2B* function, a yeast two-hybrid (Y2H) screen identified 151 potential interacting proteins. Gene Ontology (GO) enrichment analysis revealed that these interactors are mainly involved in antioxidant defense and ion transport. Further validation confirmed direct interactions between *MsRCI2B* and both calmodulin (CaM) and vacuola H⁺-ATPase (V-H⁺-ATPase), suggesting that *MsRCI2B* contributes to ion homeostasis through interactions with CaM and V-H⁺-ATPase, thereby promoting Na⁺/K⁺ balance and enhancing salt tolerance. These findings provide new insights into the role of *MsRCI2B* in salt stress responses and underscore its potential as a genetic target for enhancing salinity tolerance in forage crops.

## 1. Introduction

Soil salinization is a major global environmental challenge, adversely affecting over 20% of irrigated croplands worldwide [1,2], and it poses a significant threat to agricultural sustainability. Salinity stress impacts plants through mechanisms such as ion imbalance [3], osmotic stress [4], oxidative damage [5], and physiological disorders [6]. Under saline conditions, the accumulation of excess Na^+^ and Cl^−^ disrupts the balance of essential ions and leads to the accumulation of toxic substances [7]. Additionally, the production of reactive oxygen species (ROS) under osmotic stress damages cell membranes and photosynthetic pigments, severely affecting plant growth and health [8,9].

As an important forage crop, alfalfa (*Medicago sativa* L.) is vital for livestock productivity [10]. With the growing pressure on arable land due to soil salinization and an increasing growth population, the demand for alfalfa production is rising. Thus, there is an urgent need to develop new strategies to promote its cultivation and yield under saline-alkaline conditions [11]. Genetic engineering, through the overexpression of salt-tolerant genes, has proven effective in developing salt-tolerant plants. Transgenic research has focused on utilizing genes from various sources to enhance the salt resistance of alfalfa [12]. Our goal is to identify salt-tolerant genes and provide valuable genetic resources for breeding salt-tolerant alfalfa varieties.

The *RCI2* (Rare Cold Induced 2) gene family, characterized by the presence of the PMP_3_ domain (PF01679), is conserved across plants and homologous to the PMP_3_ protein in *Saccharomyces cerevisiae* [13]. Yeast strains deficient in PMP_3_ exhibit increased sensitivity to Na^+^, while the *AtRCI2A* gene restores Na^+^ resistance, suggesting functional similarity among *RCI2* genes [14]. However, not all *RCI2* genes can complement PMP_3_ function, indicating that different *RCI2* genes may have distinct biological roles. In addition to *Arabidopsis*, the *RCI2* gene family has been studied in other plant species, including *OsLti6A* and *OsLti6B* in rice [15], *wpi6* in wheat [16], *BLT101.1* and *BLT101.2* in barley [17], *ZmPMP3* and *ZmPMP3-6* in maize [18], and *CsRCI2A* in *Linum usitatissimum* [19]. These genes are primarily induced by cold and drought stress, but their expression is most notably elevated under salt stress, classifying them as salt tolerance genes.

In alfalfa, Overexpression of *MsRCI2A*, *MsRCI2B*, or *MsRCI2C* enhanced tolerance to both salt (200 mmol·L^−1^ NaCl) and mild alkaline (100 mmol·L^−1^ NaHCO_3_, pH 8.5) stress to varying degrees, with alkaline stress causing more severe growth inhibition than salt stress [20]. *MsRCI2D* conferred greater salt tolerance *MsRCI2E*, as evidenced by higher chlorophyll retention and lower MDA accumulation indicating reduced oxidative damage [21]. Despite their sequence homology *MsRCI2s* exhibited distinct expression patterns in response to stress, suggesting paralog-specific regulatory roles in abiotic stress adaptation.

RCI2 proteins can enhance salt stress resistance by regulating ion balance. Overexpression of *RCI2* increases K^+^ and Ca^2+^ ions concentration in plant tissues, thereby reducing Na^+^ toxicity and osmotic damage [22]. This regulation is often linked with genes involved in ion homeostasis, such as *H^+^-ATPase*, Na^+^/H^+^ transporter *SOS*, and *HKT1* [23,24]. Additionally, Ca^2+^ serves as an intracellular messenger, binding with calmodulin (CAM), which further enhances antioxidant capacity under abiotic stress [25]. While previous studies have shown that overexpression of the *MsRCI2* genes in alfalfa improve ion balance and antioxidant capacity under salt stress, the precise mechanism by which RCI2 exerts these effects remain unclear.

In this study, we employed bioinformatics methods—including genome-wide screening, phylogenetic reconstruction, and conserved motif analysis—to comprehensively identify *MsRCI2* homologs and investigate their functional divergence. These analyses were particularly critical for distinguishing paralogous genes from allelic variants within the highly complex autotetraploid genome of alfalfa. Based on the differential reponse of *RCI2* genes to abiotic stress, we further analyzed the expression of *MsRCI2* family members under salt stress. Among them, *MsRCI2B* exhibited the most significant upregulation (*p* < 0.001), prompting to investigate its specific role under NaCl treatment through functional validation. This study provides new insights into the molecular mechanisms of salt stress regulation in plants and highlights *MsRCI2B* as a promising candidate gene for improving salinity tolerance in crop species.

## 2. Result

### 2.1. MsRCI2 Gene Family Analysis

#### 2.1.1. Sequence and Phylogenetic Analysis

A total of 22 *MsRCI2* genes, including putative allelic variants, were identified and categorized into six distinct loci (Table 1). The biophysical information of all MsRCI2 proteins were calculated (Table S2). The MsRCI2B protein contain 54 amino acids, similar to the proteins in the RCI2I group (MsRCI2A–D). However, proteins in the RCI2II (MsRCI2E–F) have a larger size, ranging from 76 to 77 amino acids due to additional residues at the C-terminal (Figure S1). All MsRCI2 proteins, including MsRCI2B, have theoretical isoelectric points (pI) below 7, suggesting they are likely positively charged in their physiological state. The aliphatic index and grand average of hydropathicity values indicate that all MsRCI2 proteins are hydrophobic.

**Figure 1:**
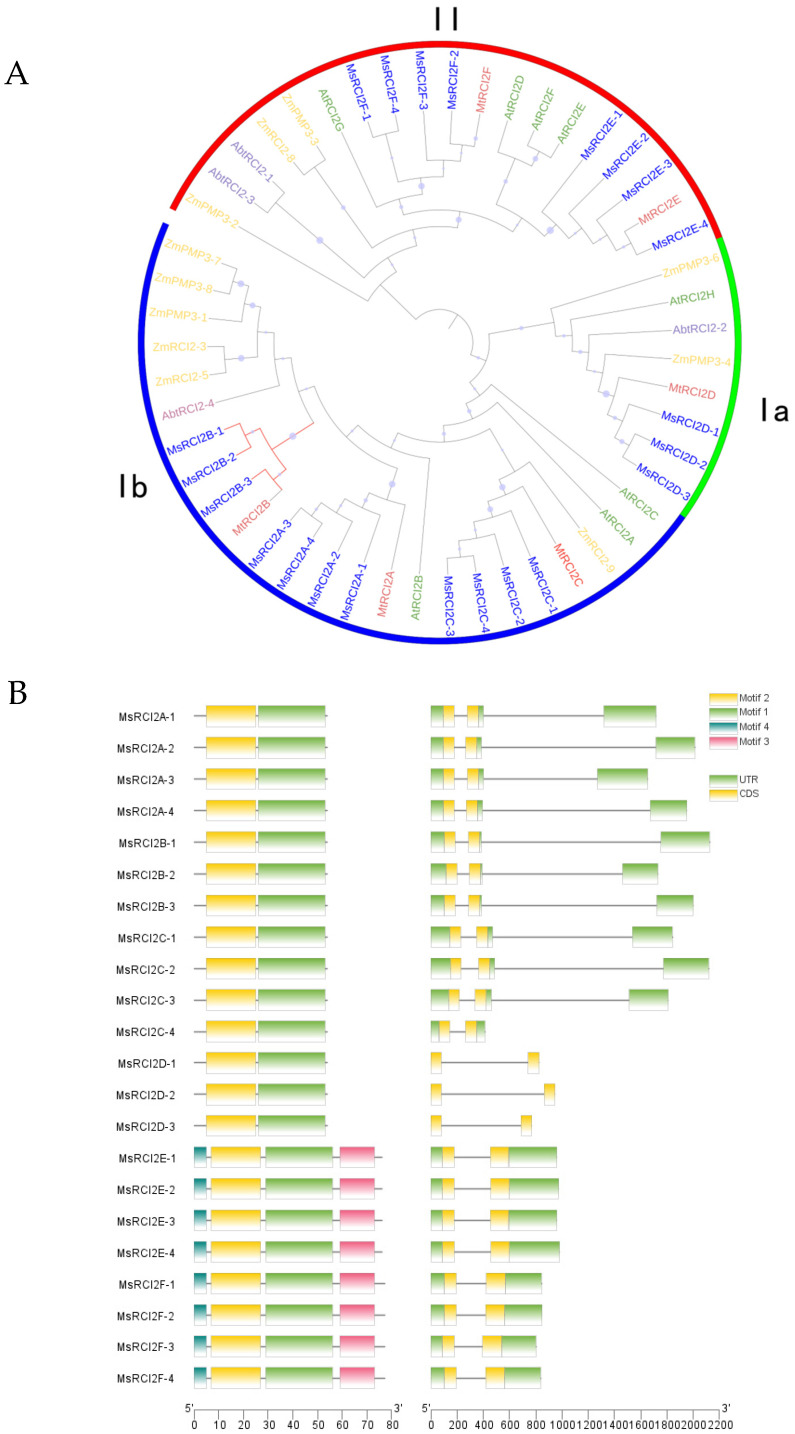
Evolution relationship of *MsRCI2* genes. (**A**) Phylogenetic tree of the *RCI2* genes family. Red: *Medicago truncatula*, Dark bule: *Medicago sativa*, Green: *Arabidopsis thaliana*, Yellow: *Zea mays*, Pale Purple: *Amborella trichopoda*. Roman numerals (Ia, Ib, II) denote major clades. Bootstrap values are indicated by dot sizes. (**B**) Gene and protein structure analysis of *MsRCI2* genes. UTR: Untranslated Regions.

To investigate the evolutionary relationships of the *MsRCI2* genes alfalfa family, a phylogenetic tree was constructed using the maximum likelihood method. The analysis included 18 alfalfa (*Medicago sativa*), 11 maize (*Zea mays*), 8 Arabidopsis (*Arabidopsis thaliana*), 8 amborella (*Amborella trichopoda*) and 6 barrel medic (*Medicago truncatula*) sequences (Figure 1A). The RCI2 family could be divided into two main groups: RCI2I and RCI2II. The RCI2II group is characterized by a larger C-terminal region compared to others. Most *RCI2* genes belong to the RCI2I group, which can be further subdivided into sub-groups Ia and Ib. Notably, *MsRCI2B* gene belongs to sub-group Ib.

In terms of motif composition, all *MsRCI2* genes contain motif 1 and 2, which are part of the PMP_3_ domain (Figure S1). However, proteins in the RCI2II group also have unique motif 3 and 4, distinguishing them from other groups (Figure 1B). The exon–intron structure of *MsRCI2* genes is the consistent, with all genes containing one intron.

#### 2.1.2. Chromosomal Localization and Collinearity Analysis

**Figure 2:**
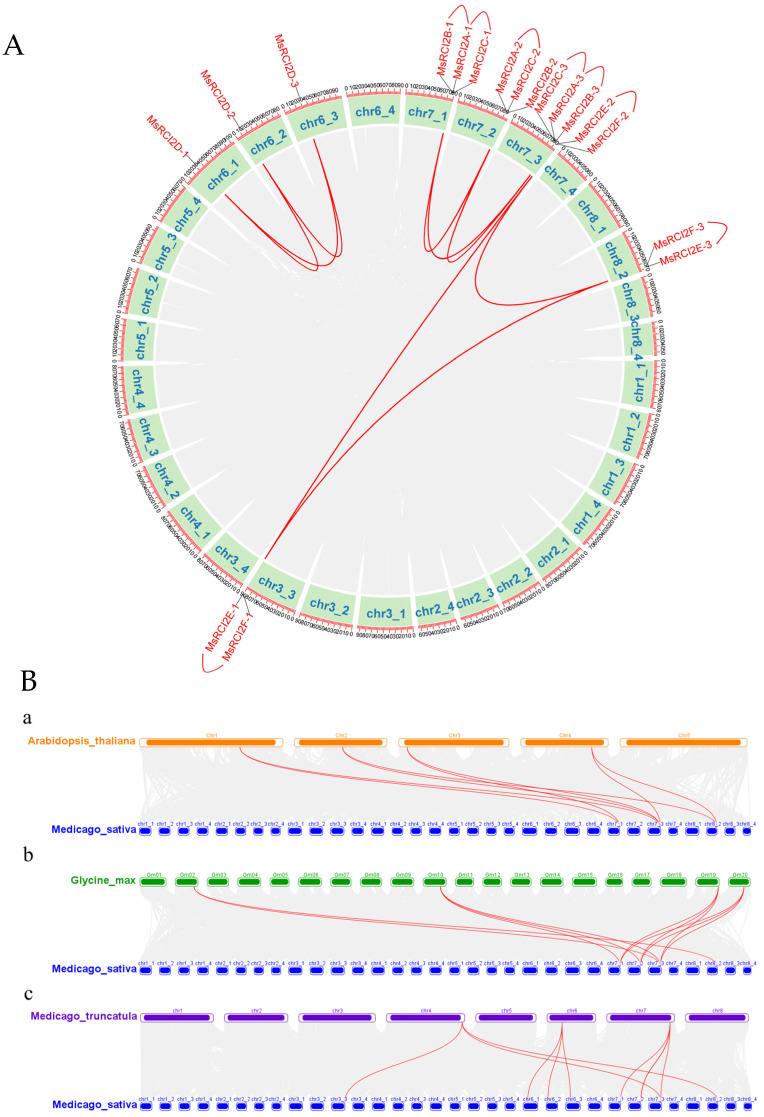
Chromosomal Localization and Collinearity Analysis of *RCI2* genes. (**A**) Collinearity analysis of *RCI2* genes within *Medicago sativa* showing gene density and pairwise collinearity (gray curve whole genome, red curve: *RCI2* genes). (**B**) Collinearity analysis between *Medicago sativa* and other species, highlighting *RCI2* gene relationships. a: the collinear relationships between *Medicago sativa* and *A. thaliana*, b: the collinear relationships between *Medicago sativa* and *G. max*, c: the collinear relationships between *Medicago sativa* and *G. max*.

Chromosomal localization and collinear analysis were performed based on *Medicago sativa* L. genome annotation (Figure 2A and Figure S1). A total of 18 *MsRCI2* genes (4 *MsRCI2s* were not assigned to chromosomes) were distributed across eight chromosomes, forming three subfamilies. Specifically, genes in sub-group Ib, to which *MsRCI2B* belongs, are clustered on the seventh chromosome, while genes in sub-group Ia are located on the sixth chromosome. The genes in the RCI2II group have a more scattered chromosomal distribution. Collinearity analysis revealed that genes and gene clusters located on different chromosomes show collinearity, with gene clusters often arising from tandem gene duplications.

We analyzed the collinear relationships between *Medicago sativa* and other species, including *M. truncatula, G. max,* and *A. thaliana* (Figure 2B). A collinear relationship was observed between *Medicago sativa* and *Medicago truncatula* in all RCI2 groups, while only group Ib and group II genes showed collinearity with *Glycine max* and *Arabidopsis thaliana*. Notably, sub-group Ib of the *MsRCI2* gene famliy demonstrated a high degree of conservation, both intraspecifically and interspecifically, indicating its evolutionary stability.

#### 2.1.3. Role of *MsRCI2B* in Salt Tolerance

**Figure 3:**
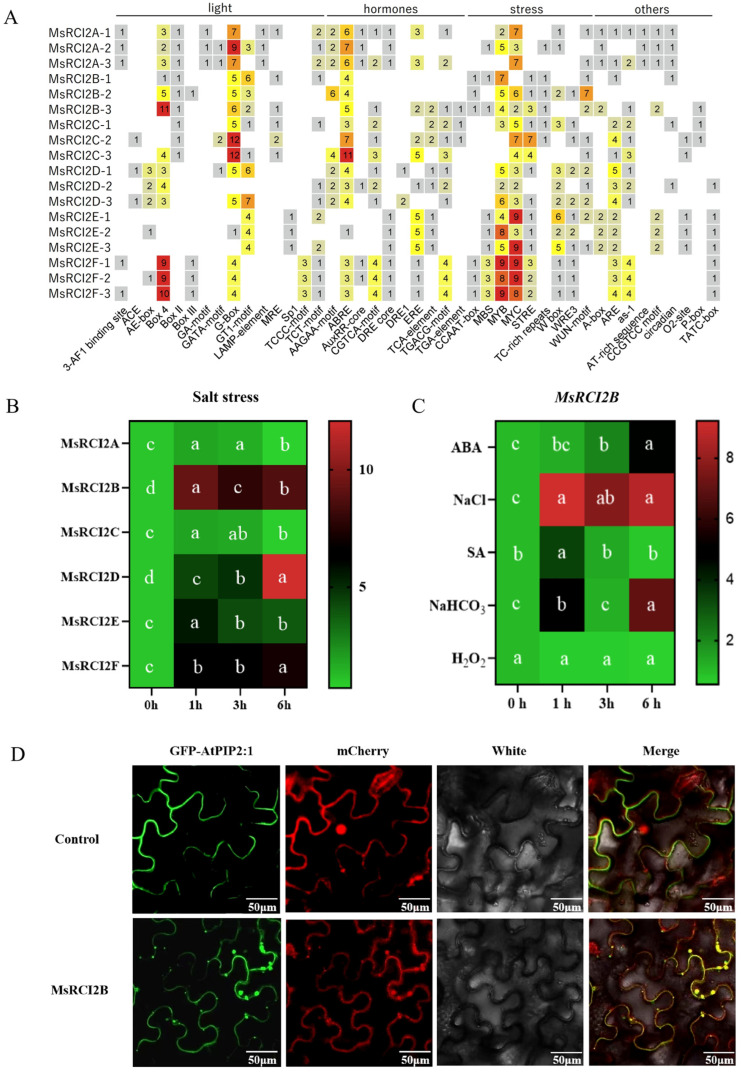
Analysis of cis-regulatory elements and expression patterns of *MsRCI2* genes. (**A**) Heatmap of cis-regulatory element analysis. The color represents the number of cis-elements possessed. (**B**) Expression heatmap of *MsRCI2* genes under salt stress (200 mM NaCl) at 0, 1, 3, and 6 h. Significance analysis was performed for different time points within the group. Different letters indicate significant differences (*p* < 0.05, one-way ANOVA with Tukey’s test) among time points within the same treatment. (**C**) Expression heatmap of *MsRCI2B* under various treatments (200 μM ABA, 100 μM SA, 200 mM NaCl, 200 mM NaHCO_3_, and 100 μM H_2_O_2_) at 0, 1, 3, and 6 h. Significance analysis was performed for different time points within the group. (**D**) Subcellular localization of MsRCI2B-mCherry fusion protein. GFP-AtPIP2;1 was used as a membrane marker, and the mCherry empty vector served as the control.

To investigate the regulatory mechanisms of *MsRCI2* genes, we analyzed cis-acting elements in their promoter regions and assessed their expression under various stress treatments. The promoters contained core cis-acting elements (Figure 3A), including several stress-responsive elements. Notably, ABA-responsive elements (AAGAA-motif) were present in all *MsRCI2* promoters, indicating that *MsRCI2B* may be highly responsive to ABA. Additional phytohormone-responsive elements, such as those responsive to ethylene (ERE), methyl jasmonate (MeJA, TGACG-motif), salicylic acid (SA, TCA-element, CGTCA-motif), and auxin (TGA-element, AuxRR-core), were also identified, though less prominently represented. Expression analysis under salt stress revealed non-significant (ns) induction of *MsRCI2A* and *MsRCI2C*, whereas *MsRCI2B* exhibited a striking response, with transcript levels increasing 8-fold (*p* < 0.001) within 1 h of treatment compared to the control. While *MsRCI2D*, *MsRCI2E*, and *MsRCI2F* also displayed salt-inducible expression, their induction levels were markedly lower than that of *MsRCI2B*. These results strongly suggest that *MsRCI2B* plays a predominant role in the early response to salt stress.

We investigated the expression of the *MsRCI2B* gene under various treatments, including 200 μmol/L ABA, 100 μmol/L SA, 200 mmol/L NaCl, 100 μmmol/L H_2_O_2_, and 200 mmol/L NaHCO_3_ treatments (Figure 3C), with peak expression occurring at 3 h post treatment. The gene was also moderately induced under salt stress, showing a delayed maximal expression at 6 h. Under ABA treatment, expression of *MsRCI2B* increased dramatically at 6 h, approximately five times higher than the control, while SA treatment resulted in a peak at 1 h with minimal changes by 6 h. No significant changes were observed under H_2_O_2_ treatment. These results suggest that *MsRCI2B* plays a key role in both salt and alkaline stress response. We investigated the subcellular localization of MsRCI2B using 35S:mCherry fusion fluorescent protein. As shown in Figure 3D, the MsRCI2B-mCherry fusion protein was exclusively localized to the plasma membrane (PM).

### 2.2. Overexpression of MsRCI2B Enhances Salt Tolerance and Antioxidant Capacity in Medicago sativa

**Figure 4:**
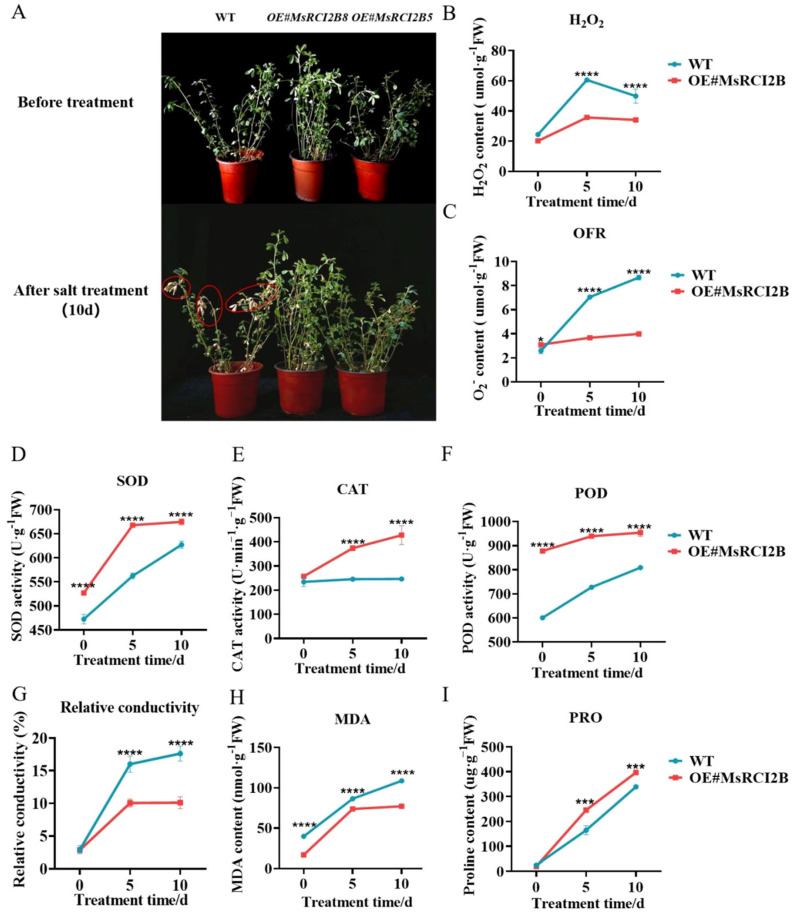
Overexpression of *MsRCI2B* enhances salt tolerance in alfalfa via osmotic adjustment and antioxidant defense. Wilted areas are marked with red circles. (**A**) The phenotype of wild−type and *OE#MsRCI2B* lines under salt stress at 0 and 10 days. (**B**,**C**) Variations in H_2_O_2_, O_2_^−^ in *MsRCI2B*−overexpressing alfalfa leaves subjected salt stress. (**D**–**F**) Activity levels of SOD, POD, and CAT enzymes. (**G**) Relative conductivity of WT and *OE#MsRCI2B* lines. (**H**,**I**) MDA content and proline content of WT and *OE#MsRCI2B* lines. Statistical significance was assessed using Tukey’s test to compare *MsRCI2B*−overexpressing lines with wild-type (WT) controls at time points (0, 5, and 10 h post-treatment). Significance thresholds are denoted as: *** *p* < 0.001, **** *p* < 0.0001. Error bars indicate standard deviation (SD) derived from three independent biological replicates, with each experiment including three technical replicates to ensure measurement consistency.

To further investigate the effects of *MsRCI2B* on alfalfa growth and salt tolerance, we generated transgenic alfalfa Ms*RCI2B*-overexpressing lines (OE#*RCI2B8*, OE#*RCI2B5*). These lines exhibited significantly higher expression levels of the *MsRCI2B* gene than the wild type (*p* < 0.001). Colocalization of mCherry fluorescence in roots confirmed successful gene expression (Figure S4). After 10 days of treatment with 200 mmol/L NaCl, the transgenic lines maintained better growth and exhibited fewer yellowed leaves (Figure 4A), while wild-type (WT) growth exhibited inhibited growth, characterized by leaf wilting and near death.

To assess oxidative damage under salt stress, we measured the accumulation of hydrogen peroxide (H_2_O_2_; Figure 4B) and superoxide anion (O_2_^−^; Figure 4C). The *OE#RCI2B* lines showed lower H_2_O_2_ levels compared to the WT. After salt treatment, both H_2_O_2_ and O_2_^−^ levels were significantly decreased in the *MsRCI2B*-overexpressing alfalfa compared to WT plants (*p* < 0.0001). To investigate the underlying mechanism, we have further analyzed the activities of key antioxidant enzymes, including peroxidase (POD), superoxide dismutase (SOD) and catalase (CAT). In the absence of salt stress, transgenic lines showed higher baseline SOD and POD activities. Upon salt treatment, SOD, POD, and CAT activities were significantly elevated in the *OE#RCI2B* lines (*p* < 0.0001) (Figure 4D–F).

To further elucidate the mechanisms underlying salt tolerance in transgenic alfalfa, we examined relative conductivity, malondialdehyde (MDA) content, and proline levels. Under salt stress, OE lines exhibited significantly lower electrolyte leakage and MDA levels, while proline content was significantly elevated compared to WT plants at both 5 and 10 days of treatment (*p* < 0.0001) (Figure 4G−I). These results suggest that overexpression of *MsRCI2B* enhances antioxidant defense, reduces oxidative damage, and contributes to improved cellular stability and salt tolerance under salt stress in alfalfa.

### 2.3. MsRCI2B Modulates Ion Homeostasis Under Salt Stress

**Figure 5:**
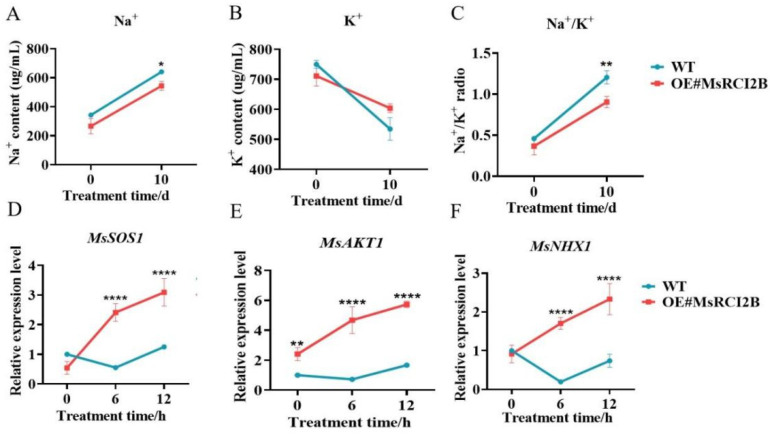
Changes in sodium and potassium content and differential expression of ion transport related genes in *OE#MsRCI2B* plants under salt stress. (**A**) Na^+^ content in the leaves of *OE#MsRCI2B* plants. (**B**) K^+^ content in the leaves of *OE#MsRCI2B* plants. (**C**) Na^+^/K^+^ ratios in the leaves of *OE#MsRCI2B* plants. (**D**–**F**) Expression levels of *MsSOS1*, *MsAKT1*, and *MsNHX1* gene in the leaves of transgenic plants as measured by qPCR. Statistical significance was assessed using Tukey’s test to compare *MsRCI2B*-overexpressing lines with wild-type (WT) controls at time points (0, 5, and 10 h post treatment). Significance thresholds are denoted as * *p* < 0.05, ** *p* < 0.01, **** *p* < 0.0001. Error bars indicate standard deviation (SD) derived from three independent biological replicates, with each experiment including three technical replicates to ensure measurement consistency.

To elucidate the regulatory role of *MsRCI2B* in maintaining ion homeostasis under salt stress, we measured the Na^+^ content and K^+^ content in the leaves of transgenic and WT lines. After 10 days of treatment with 200 mmol·L^−1^ NaCl, both WT and transgenic alfalfa showed a reduction in K^+^ content, an increase in Na^+^ content, and a higher Na^+^/K^+^ ratios, with transgenic plants showing higher Na⁺/K⁺ ratios compared to wild-type controls (*p* < 0.01) (Figure 5A–C).

To gain further insights into the molecular mechanisms responsible for these changes in ion homeostasis, we analyzed the expression of key ion transporter genes—*MsSOS1*, *MsAKT1*, and *MsNHX1*. *MsSOS1*, a plasma membrane Na^+^/H^+^ antiporter, was significantly upregulated in the OE#*MsRCI2B* lines compared to WT under salt stress (*p* < 0.0001) (Figure 5D). *MsAKT1*, which encodes a K⁺ channel responsible for K⁺ uptake, also showed enhanced expression in transgenic plants (*p* < 0.0001), indicating that efficient K^+^ uptake and transport may contribute to a lower Na^+^/K^+^ ratio (Figure 5E). *MsNHX1*, a vacuolar Na^+^/H^+^ antiporter, the expression level was significantly upregulated in the *OE#MsRCI2B* line (Figure 5F). These findings demonstrate that the overexpression of *MsRCI2B* coordinately regulates plasma membrane Na⁺ extrusion (*MsSOS1*), K⁺ acquisition (*MsAKT1*), and vacuolar Na⁺ compartmentalization (*MsNHX1*), and they were was also markedly upregulated in OE lines relative to WT under salinity (*p* < 0.0001).

### 2.4. Screening and Functional Clustering (GO) Analysis of Candidate Interacting Proteins

The plasmid pBT3-N-*MsRCI2B* was successfully expressed (Figure S3A) in the yeast two-hybrid (Y2H) system and exhibited no self-activation ability (Figure S3B), meeting the requirements for library screening. Candidate interacting proteins were selected and cultured on quadruple-deficient (QDO) medium to assess their growth (Figure S3C). X-gal staining was then added to the medium to verify β-galactosidase activity, and blue coloration confirmed positive interactions (Figure S3D).

PCR-positive clones were sequenced and subjected to BLAST analysis (NCBI-blast-2.14.0+), resulting in the identification of 151 putative MsRCI2B-interacting proteins. The gene functional clustering (GO) analysis of these 151 proteins was performed using PANTHER version 17.0.

**Figure 6:**
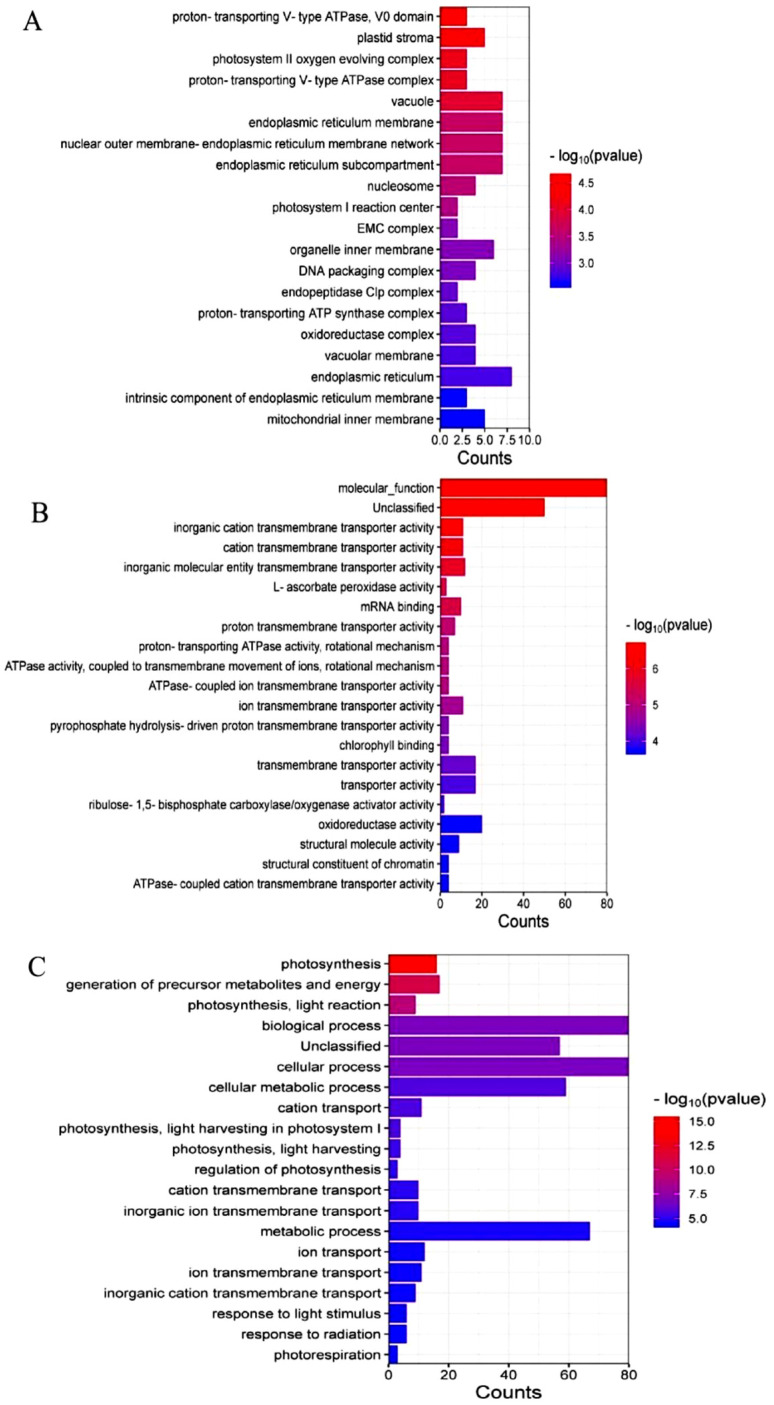
GO analysis of screened genes. (**A**) GO−CC (Cell Component) analysis of screened genes. (**B**) GO−BP (Biological Process) analysis of screened genes. (**C**) GO−MF (Molecular Function) analysis of screened gene.

Cellular component analysis showed distinct compartmentalization patterns of MsRCI2B-interacting proteins, with predominant enrichment in the endoplasmic reticulum, membrane systems, and the internal environment of the organelles (Figure 6A), highlighting their critical involvement in organellar communication and membrane-mediated cellular processes (Figure 6A). Molecular function analysis showed that these proteins were mainly involved in transmembrane ion transport and oxidoreductase activity, suggesting roles in maintaining ionic and redox balance (Figure 6B). Furthermore, biological process analysis highlighted significant enrichment in pathways related to cellular metabolic homeostasis, photosynthesis, and ion transmembrane trafficking (Figure 6C). These findings suggest that MsRCI2B-associated proteins form a functional network regulating essential cellular activities, particularly through coordinated modulation of ion homeostasis, redox balance, and energy conversion processes.

### 2.5. Expression Analysis of Candidate MsRCI2B-Interacting Protein Genes

The above results indicate that these interacting proteins are mainly involved in biological processes such as ion transport (Table S2), transmembrane transport (Table S3), photosynthesis (Table S4), and enzyme proteins (Table S5). The protein transmembrane structure, subcellular localization, and physicochemical properties of candidate interacting proteins were analyzed.

**Figure 7:**
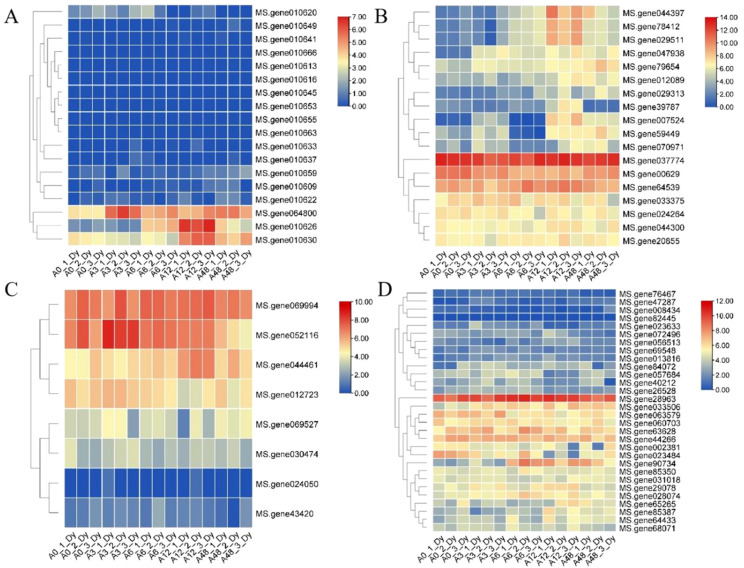
Transcriptome analysis of candidate proteins in alfalfa under salt stress at 0, 3, 6, 12, 48 h. The heatmap was generated using TBtools and the data were normalized. Lines: Cluster genes with similar expression levels. (**A**) Heatmap of candidate proteins associated with photosynthetic reactions. (**B**) Heatmap of candidate interacting proteins associated with ion transport. (**C**) Heatmap of candidate interacting proteins associated with transmembrane transport. (**D**) Heatmap of candidate interacting proteins associated with enzyme.

To explore their transcriptional responses under salt stress, heatmaps were generated using the “DaYe” transcriptome data under salt stress. Among the MsRCI2B-interacting proteins, several photosynthesis-related proteins were identified, with majority of these candidate interacting proteins being downregulated (Figure 7A). Notably, The expression of plastocyanin (*MS.gene064800*) showed elevated expression at 3, 6, 12, and 48 h post treatment, with the highest expression observed at 3 h during the early stage. Additionally, the oxygen-evolving enhancer proteins of photosystem II (*MS.gene010626*, *MS.gene010630*) exhibited peak upregulation at 12 h post treatment. These findings suggest that the salt tolerance function of *MsRCI2B* may involve regulation of Photosystem II and antioxidant defense pathways.

Among the proteins that MsRCI2B interacts with, the ion transport class includes 22 protein, which involve ATP synthases, ion pumps, and the transport of metal ions (zinc, iron, aluminum, etc.). Most of these genes were transcriptionally upregulated under salt stress (Figure 7B). Three V-type H^+^-ATPases (*MS.gene012089*, *MS.gene59449*, *MS.gene012089*) showed increased expression at 12 and 48 h, indicating a role in proton transport and cellular ion balance. In addition, calmodulin (*MS.gene39787*) is initially downregulated but later showed upregulation at 12 h, suggesting a possible regulatory role in signal transduction during salt stress.

Additionally, several interacting proteins were associated with transmembrane transport of small molecules, including inositol, oligopeptides, and sugars—key processes for intracellular transport and metabolic regulation. Nearly all of these proteins were upregulated (Figure 7C), with the ABC transporter B family protein (*MS.gene044461*) reaching peak expression at 12 h. A transmembrane protein (*MS.gene069994*) was significantly more up-regulated compared to the other proteins.

A large proportion of MsRCI2B-interacting proteins were enzymes, including oxidoreductases, transferases, hydrolases, synthetases, and isomerases, all of which play crucial roles in metabolic regulation. Most redox-related enzymes were downregulated (Figure 7D), suggesting that *MsRCI2B* may mitigate salt stress through the scavenging of reactive oxygen species. Moreover, the peptidyl-prolyl cis-trans isomerase (*MS.gene28963*) exhibited a notably higher level of upregulation compared to the other proteins.

### 2.6. Further Verification of Candidate Interacting Proteins with MsRCI2B

**Figure 8:**
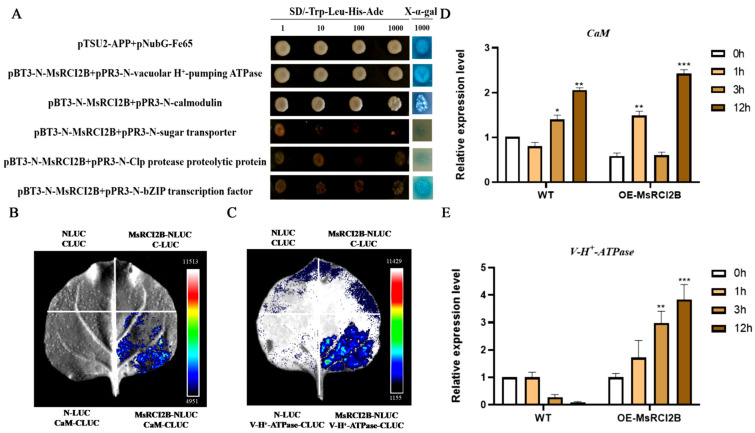
Validation of MsRCI2B interacting protein. (**A**) Validation of MsRCI2B-interacting protein by yeast two-hybrid. (**B**) The interaction between MsRCI2B and CaM was verified by the Dual-LUC assay. Images are color coded according to the colored bars (4951 to 15,513) shown on the right side. (**C**) The interaction between MsRCI2B and V-H^+^-ATPase was verified by Dual-LUC assay. Images are color coded according to the colored bars (1155 to 11,429) shown on the right side. (**D**) Analysis of calmodulin in response to salt stress in *MsRCI2B*-overexpressing alfalfa in comparison with the wild-type plants. (**E**) Analysis of vacuolar V-H^+^-ATPase in response to salt stress in *MsRCI2B*-overexpressing alfalfa in comparison with the wild-type plants (* *p* < 0.05, ** *p* < 0.01, *** *p* < 0.001). Error bars indicate standard deviation (SD) derived from three independent biological replicates, with each experiment including three technical replicates to ensure measurement consistency.

Given the central roles of ion toxicity (e.g., Na⁺ accumulation) and oxidative stress in salt-stress responses, we prioritized candidate proteins associated with ion transport and redox homeostasis. From the Y2H screening, candidates were prioritized based on their repeated identification in independent assays and functional linkage to salt-stress responses. To further validate the interaction between MsRCI2B and five full-length positive clones, including the V-type proton pump, CaM, clp hydrolyzed protein, sugar transporter and bZIP. To validate these interactions, full-length coding sequences of the five candidate proteins were cloned into the pPR3-N vector. Yeast two-hybrid assays confirmed interactions between MsRCI2B and the V-type proton pump as well as CaM (Figure 8A). These two interactions were further verified using Dual-Luciferase Complementation assays, which confirmed the physical interaction of MsRCI2B with both CaM (Figure 8B) and the V-type proton pump (Figure 8C).

To further investigate the roles of these interacting proteins under stress and their relationship with MsRCI2B, we measured the expression patterns of CaM (Figure 8D) and the V-type proton pump (Figure 8E) in wild-type and *MsRCI2B*-overexpressing alfalfa under salt stress (200 mM NaCl) at 0, 1, 3, and 12 h. In wild-type plants, the expression of *CaM* significantly upregulated at 3 and 12 h of salt treatment. The expression pattern of *CaM* followed a distinct pattern-rising at 1 h, declining at 3 h, and reaching its highest level at 12 h in *MsRCI2B*-overexpressing alfalfa. Additionally, the expression of the *V-H^+^ATPase* was significantly elevated in *MsRCI2B*-overexpressing alfalfa after 12 h of salt treatment, followed by an increase at 3 h, compared to wild-type plants. These results suggest that *MsRCI2B* plays a crucial role in modulating ion transport and stress response, particularly under prolonged salt stress.

## 3. Discussion

### 3.1. Identification and Evolutionary Characteristics of the MsRCI2 Gene Family

The *RCI2* (Rare Cold-Inducible 2) gene family encodes small hydrophobic proteins that are evolutionarily conserved and play critical roles in regulating ion homeostasis, stabilizing membranes, and enhancing tolerance to abiotic stress in plants [23]. In this study, we identified six members of the *MsRCI2* family in *Medicago sativa*, all of which contain the highly conserved PMP_3_ domain, consistent with previous findings in other plant species. Collinearity analysis between *M. sativa* and *M. truncatula* revealed a high degree of evolutionary conservation, suggesting potential functional redundancy or diversification among *MsRCI2* genes.

We also found that the *MsRCI2* family contains numerous tandem repeat gene pairs, with both the RCI2Ib and RCI2II subfamilies forming gene clusters. This genomic architecture provides evidence that tandem duplication events have driven the evolutionary expansion of these subfamilies. Motif analysis further revealed that the proteins in the RCI2II subfamily possess unique motifs at both the N- and C-terminal, which may contribute to functional diversification. These findings are consistent with studies in other species, where C-terminal sequence divergence serves as a key differentiating feature among RCI2 subfamily members [26]. However, the functional roles of the N-terminal motifs in MsRCI2 from subfamily II warrant further investigation.

### 3.2. Functional Role of MsRCI2B in Salt Stress Tolerance

Cis-acting element analysis of the *MsRCI2* promoters revealed the presence of stress-responsive motifs, supporting the notion that *MsRCI2* genes are involved in stress responses. Expression analysis under salt stress conditions showed differential responses among the *MsRCI2* family members, with *MsRCI2B* being the most highly upregulated under salinity stress, suggesting that *MsRCI2B* may have undergone evolutionary specialization through subfunctionalization. Similar findings have been reported in *Arabidopsis* [27,28] and *Triticum aestivum* [29], where specific *RCI2* genes are highly inducible under abiotic stress conditions.

*MsRCI2B*, a member of the conserved RCI2 family, plays a key role in plant adaptation to multiple abiotic stresses, including salinity and extreme temperatures [12]. Localized to the plasma membrane, *MsRCI2B* regulates cellular functions related to membrane stability, ion homeostasis, and oxidative stress, which are essential for plant survival under abiotic stress conditions [30].

To explore the molecular mechanism underlying *MsRCI2B*-mediated salt tolerance, we conducted yeast two-hybrid (Y2H) screening, identifying 151 potential interacting proteins. GO enrichment analysis revealed that many of these interactors are involved in antioxidant defense, including NADPH-dependent quinone oxidoreductase, leaf ferredoxin-NADP reductase and thylakoid-bound ascorbate peroxidase. We hypothesize that interactions may play a critical role in mitigating oxidative stress.

Oxidoreductases, including those mediating the non-phosphorylating alternative respiratory pathway (via NAD(P)H dehydrogenase), are vital for stress responses [31]. These enzymes help uncouple carbon metabolism from adenylate control, reducing the formation of reactive oxygen species (ROS), which can cause cellular damage under salt stress. Antioxidant enzymes, through ROS scavenging, are essential for maintaining cellular integrity during stress. Our results showed that *MsRCI2B*-overexpressing alfalfa had significantly higher antioxidant enzyme activity (SOD, POD, and CAT) (Figure 4D–F) and lower oxidative damage markers (MDA, H_2_O_2_, and O_2_^−^) (Figure 4G–I) compared to non-transgenic controls. These findings suggest that *MsRCI2B* may help manage oxidative stress management by enhancing ROS-scavenging enzymes activity.

In addition, our analysis revealed that many of proteins identified in the screening library are associated with ion transport and transmembrane transport, including ATP synthase, V-type proton pumps, aluminum-induced proteins, and calmodulin. Ion homeostasis analysis showed that *MsRCI2B*-overexpressing plants maintained a lower Na^+^/K^+^ ratio than wild-type plants under salt stress, suggesting enhanced sodium exclusion and potassium retention. This is further supported by the upregulation of key ion transporters such as *MsSOS1* [32], *MsAKT1* [33], and *MsNHX1* [34] (Figure 5D–F), which facilitate Na^+^ efflux and K^+^ uptake, maintaining ionic balance under salinity stress. These results highlight the crucial role of *MsRCI2B* in regulating ion transport and maintaining cellular ionic balance.

### 3.3. MsRCI2B Interacts with CaM and V-H^+^-ATPase to Modulate Salt Stress Response

Protein–protein interaction validation identified calmodulin (CaM) and vacuolar V-H^+^-ATPase as key interactors with MsRCI2B, confirmed through both yeast dual-hybrid experiments (Figure 8A) and dual-luciferase assays (Figure 8B,C). CaM is a major calcium sensor protein conserved across eukaryotes [35] and plays critical roles in mediating calcium (Ca^2+^)-dependent signaling pathways in response to various abiotic stresses, including heat, cold, salt, drought, and toxic metals [36,37,38,39]. Upon exposure to stress, plants activate Ca^2+^ pumps and ion channels [40,41,42], initiating a signaling cascade. The interaction between MsRCI2B and CaM suggests that *MsRCI2B* may be involved in Ca^2+^-dependent signaling pathways to modulate salt stress responses.

Additionally, the interaction between MsRCI2B and V-H^+^-ATPase suggests a MsRCI2B plays a role in maintaining proton gradients and membrane potential, which are essential for Na^+^/H^+^ exchange and intracellular pH regulation under salt stress [43]. V-H^+^-ATPases are known to facilitate vacuolar Na^+^ sequestration, reducing cytosolic Na^+^ toxicity in salt-stressed plants. Our findings further support that MsRCI2B functions through both signaling (via CaM) and ion transport (via V-H⁺-ATPase) mechanisms to enhance salt stress tolerance.

### 3.4. Proposed Model of MsRCI2B Function in Salt Stress and Agricultural Implications

**Figure 9:**
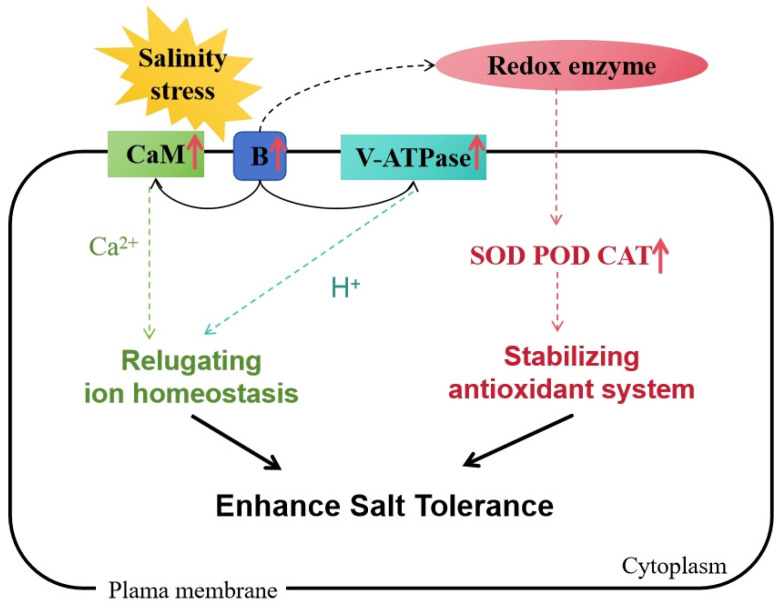
Model of *MsRCI2B* gene response to salinity stress. This model illustrates the interactions of MsRCI2B with CaM, V-H^+^-ATPase, and redox enzyme to enhance salt tolerance by regulating ion homeostasis and ROS scavenging. Red arrows indicate upregulation.

Based on our findings, we propose a model in which *MsRCI2B* enhances salt tolerance in *Medicago sativa* by modulating ion homeostasis and oxidative stress defense (Figure 9). Under salinity stress, MsRCI2B interacts with CaM and H^+^-ATPase to regulate stress signaling and improve ion homeostasis, and it interacts with redox enzymes to clear ROS and manage oxidative stress. These findings provide valuable insights into the molecular mechanisms by which *MsRCI2B* confers salt tolerance, positioning it as a potential target for genetic improvement in crops for enhanced salt stress resistance.

## 4. Conclusions

This study demonstrates that overexpression of *MsRCI2B* enhances salt tolerance in *Medicago sativa* by regulating ion homeostasis—through interactions with CaM and V-H^+^-ATPase—and by facilitating ROS scavenging via interactions with oxidase. *MsRCI2B* modulates membrane-localized ion transporters (*MsNHX*, *MsAKT*, *MsSOS*), stabilizes antioxidant systems, and maintains ion balance under salt stress.

## 5. Materials and Methods

### 5.1. Identification of the RCI2 Family in Alfalfa

The *Medicago sativa* genome sequence and annotation data were obtained from the Genome Warehouse (GWH) at the National Genomics Data Center (NGDC), Beijing Institute of Genomics, Chinese Academy of Sciences/China National Center for Bioinformation (https://ngdc.cncb.ac.cn/gwh/, accessed on 10 Decemember 2022). The *A. thaliana* genome and annotation information were retrieved from the TAIR database, version 10 (TAIR 10) (https://www.arabidopsis.org/, accessed on 11 Decemember 2022). *Glycine max* genome (the Wm82.a2.v1 version) and its annotation file were obtained from Phytozome (https://phytozome-next.jgi.doe.gov/, accessed on 12 Decemember 2022). And the genome and annotation information of *Medicago truncatula* were sourced from the *Medicago truncatula* Genome Database (http://www.medicagogenome.org/, accessed on 12 Decemember 2022) [44].

The identification of *MsRCI2* genes was carried out using three methods: first, the protein sequences of AtRCI2s were used as probes in a BLASTp search against the alfalfa proteome to identify candidate members of the *MsRCI2* family, based on data from Joaquín Medina’s research. Second, the Hidden Markov Model (HMM) profiles of the RCI2_dimer domain PMP_3_ (PF01679) were downloaded from the Pfam database (http://pfam.xfam.org/, accessed on 13 Decemember 2022) to search for the RCI2_dimer domain from the alfalfa proteome using an e-value cut off of 1 × 10^−5^. Moreover, to predict the biophysical properties of RAD genes, ExPASy-ProtParam tool (https://web.expasy.org/protparam/, accessed on 15 Decemember 2022) was employed. Subcellular localization of the proteins was predicted using WoLF PSORT (https://wolfpsort.hgc.jp/, accessed on 16 Decemember 2022).

### 5.2. Phylogenetic Analysis, Conserved Motif, and Cis-Element Analysis of the MsRCI2 Gene Family

To investigate the phylogenetic relationships and predict the potential biological functions of *MsRCI2s*, a phylogenetic tree was constructed using the full-length RCI2s proteins of *M.sativa*, *A. thaliana* and *M. truncatula*, which by the maximum likelihood (ML) method and Le_Gascuel_2008 model [45]. with 1000 bootstrap replicates through MEGA10 software [46]. The graphical representation of the tree was further refined using iTOL [47].

Conserved motifs and domains of *MsRCI2* genes were predicted by MEME (https://meme-suite.org/meme/tools/meme, accessed on 20 Decemember 2022). The optimized parameters of MEME were set as follows: the optimum width of each motif ranged from 6 to 50, and a maximum of 10 motifs were identified. In addition, TBtools (https://github.com/CJ-Chen/TBtools, accessed on 21 Decemember 2022) [48]. was used to visualize the gene structure and identify the exon/intron boundaries. The Plant CARE database (http://bioinformatics.psb.ugent.be/webtools/plantcare/html/, accessed on 23 Decemember 2022) was utilized to predict and analyze the cis elements within the 2000 bp upstream sequences of each *MsRCI2* gene.

### 5.3. Construction of Alfalfa cDNA Library

Wild alfalfa growing for 21 days was treated with 200 mmol/L NaCl for 0, 1, 3, 12 h. Total RNA was extracted from the leaves of wild-type and transgenic alfalfa lines. Total RNA was isolated from alfalfa leaves using TRIzol^®^ Reagent, following the manufacturer’s protocol (Quanshijin, Bejing, China). RNA integrity was verified via 1.5% agarose gel electrophoresis, and purity (A260/A280 ratio > 1.8) was confirmed using a NanoDrop 2000 spectrophotometer (NanoDrop, Wilmington, NC, USA). The first strand cDNA was immediately synthesized using the reverse transcription kit (Vazyme, Nanjing, China). Each experiment includes three technical and independent biological replicates to ensure measurement consistency.

### 5.4. Gene Cloning and Decoy Expression Vector Construction

Using alfalfa cDNA as template, specific primers were designed based on the sequence of *MsRCI2B* gene (Medtr7g111450) to amplify its full-length fragment. The homologous arm primers were then designed based on the full-length fragment of *MsRCI2B*, and the target DNA bands with the expected size were excised from the gel, purified, and ligated into the yeast vector pBT3-N, which had been digested with *SfiI* restriction enzyme. The vector pBT3-N was connected to the target fragment by homologous recombination, and transformed into *Escherichia coli DH5α*-receptive cells, which were coated on ampicillin-resistant LB medium and cultured overnight at 37 °C. The pBT3-N-*MsRCI2B* decoy vector was successfully constructed and stored at −80 °C.

### 5.5. Detection and Functional Verification of Decoy Protein Self-Activation Activity

The plasmid combinations pBT3-N-*MsRCI2B* + pPR3-N (experimental group 1), pBT3-N-*MsRCI2B* + pOst1-NubI (experimental group 2), pTSU2-APP + pPR3-N (negative control), and pTSU2-APP + pNubG-Fe65 (positive control) were transferred to NMY51 yeast-receptive cells and coated with 100 μL on SD/-Trp-Leu, SD/-Trp-Leu-His, SD/-Trp-Leu-His-Ade defective solid medium. The plates were sealed and incubated at 30 °C for 3–5 days. The growth of colonies was observed to determine if the decoy protein exhibited self-activation activity.

### 5.6. Screening of Yeast Two-Hybrid Library with Membrane System

Yeast strain NMY51, carrying the pBT3-N-*MsRCI2B* decoy vector, was used as the recipient strain to prepare a receptive state. The library plasmids were transformed into the yeast cells, and the transformed cells were plated on SD/-Trp-Leu-His-deficient solid media. The plates were incubated at 30 °C for 3–5 days, and colonies with good growth were selected. These colonies were suspended in 50 μL ddH_2_O, and 10 μL drops were plated onto SD/-Trp-Leu-His-Ade-deficient media, followed by incubation at 30 °C for 3–5 days. Single colonies with good growth were selected, diluted (10^−1^ and 10^−2^), and transferred to SD/-Trp-Leu-His-Ade media containing X-gal. The plates were incubated at 30 °C for 3–5 days, and colony growth and color development were analyzed.

### 5.7. Positive Transformant Sequencing and Bioinformatics Analysis

Positive yeast colonies were selected and suspended in 50 μL ddH_2_O. Yeast liquid was used as the template for PCR amplification with pPR3-N-F universal primers. The PCR products were analyzed by agarose gel electrophoresis, and the bands were excised and sequenced by Kumei Company (Changchun, China). The primers used are listed in Supplementary Table S6. Sequencing results were analyzed using BLAST, and protein structure prediction, subcellular localization, and analysis of the physical and chemical properties were conducted using TMHMM software (http://www.cbs.dtu.dk/services/TMHMM/, accessed on 3 July 2024), PSORT software (http://psort.nibb.ac.jp, accessed on 3 July 2024), and ExPASy ProtParam (https://web.expasy.org/protparam, accessed on 4 July 2024). Additionally, gene functional clustering (GO) analysis was performed using the PANTHER version 17.0 online tool. The PANTHER Overrepresentation Test was used, with alfalfa as the reference genome.

### 5.8. Analysis of Expression of Candidate Interacting Protein Genes

Transcriptome sequencing analysis was performed on wild-type alfalfa (DaYe) subjected to salt stress (200 mM NaCl) at 0 h, 3 h, 12 h, and 48 h. Transcriptome data for the interacting protein genes were extracted, and an expression heatmap was generated using the FPKM values (log2-transformed) of gene expression at each time point, with Tbtools (v2.14.2) software.

### 5.9. Cloning of Full-Length Genes and In Vivo Interactions in Yeast

Using alfalfa cDNA as template, the full-length gene of the positive interclone was cloned. PCR products were connected with pPR3-N-vector to transform *Escherichia coli* and construct a recombinant plasmid pPR3-gene. The recombinant plasmid pPR3-gene and pBT3-N-*RCI2B* were co-transformed into yeast NMY51 and activated the positive clone. The bacterial solution was diluted 10^−1^, 10^−2^, and 10^−3^, and 2.5 μL of each dilution was plated onto SD/-Trp-Leu-His-Ade-deficient medium. After PCR verification, the diluted bacterial solution (10^−3^) was plated on SD/-Trp-Leu-His-Ade-X-α-Gal plates and incubated at 30 °C for 3–5 days. Colony growth and color development were observed.

### 5.10. RNA Extraction, cDNA Synthesis, and Quantitative Real-Time PCR (RT-qPCR)

Total RNA was extracted from the leaves of wild-type and transgenic alfalfa lines using the Plant RNAprep Pure Kit (Kangwei, Bejing, China) according to the manufacturer’s protocol. RNA quality was assessed using 2.0% agarose gel electrophoresis, and RNA concentration was determined using a NanoDrop ND-1000 spectrophotometer (NanoDrop, Wilmington, USA). The first strand cDNA was synthesized using the reverse transcription kit (Vazyme, China). Gene-specific primers were designed using PrimerBLAST (https://www.ncbi.nlm.nih.gov/tools/primer-blast/, accessed on 16 Decemember 2024) and are listed in Supplementary Table S6. RT-qPCR was performed in a 96-well (10 μL) format using Trans Start Top Green qPCR SuperMix (Vazyme Biotech, Nanjing, China), with *GAPDH* as an internal standard control. The relative expression levels were calculated using the 2^−ΔΔCT^ method with three biological replicates per sample to ensure statistical significance.

### 5.11. LCI (Luciferase Complementation Imaging)

The coding sequence of the target gene was fused with cLUC on the vector pCAMBIA-cLUC, while the *MsRCI2B* sequence was fused with nLUC on the vector pCAMBIA-nLUC. *Agrobacterium tumefasciens* GV3101 harboring 35S:V-*H^+^-ATPase* /*CaM*:-cLUC and 35S: *MsRCI2B*:-nLUC were used to co-transform leaves of 1-month-old *Nicotiana benthamiana* plants.

### 5.12. Evaluation of Salt Tolerance

Wild-type (WT) and transgenic plants were treated with 200 mM NaCl solution. To evaluate salt tolerance, relative conductivity, proline (Pro), soluble sugar, and malondialdehyde (MDA) levels were measured. Relative conductivity, MDA, proline, soluble sugar contents, antioxidant enzyme activities (SOD, POD, CAT), and levels of H_2_O_2_ and O_2_^−^ were measured using physiological kits (Suzhou Keming Biotechnology, Suzhou, China) and a UV-visible spectrophotometer. DAB and NBT staining were used to visualize reactive oxygen species (ROS) accumulation. Leaves were vacuum infiltrated in the staining solution for 30 min, stained overnight, and then decolorized in a solution (ethanol:acetic acid:glycerol = 3:1:1) and heated in a boiling water bath until all chlorophyll was removed.

### 5.13. Determination of Na^+^ and K^+^ Content

To prepare standard solutions, 1.907 g of KCl and 0.6355 g of NaCl were dissolved in deionized water to a final volume of 1 L. WT and transgenic alfalfa leaves and roots were dried at 105 °C, followed by drying at 80 °C to constant weight. A 0.05 g sample from each dried plant material was weighed. The extraction solution was prepared by mixing 5 mL concentrated nitric acid, 1 mL of 60% trichloroacetic acid, and 0.5 mL concentrated sulfuric acid. This solution was added to the sample and extracted in a 90 °C water bath. After filtration, the solution was analyzed using flame atomic absorption spectrometry.

### 5.14. Subcellular Localization Analysis

The p2300-mCherry vector was digested with *BamH* I and *Kpn* I restriction enzymes. The target fragment was ligated into the linearized vector using Vazyme’s one-step cloning kit and subsequently transformed into *E. coli* DH5α competent cells. Positive clones were verified by Sanger sequencing and transformed into *Agrobacterium tumefaciens* strain GV3101. Bacterial suspensions were prepared in infiltration buffer (10 mM MgCl_2_, 10 mM MES, 150 μM acetosyringone, pH 5.7) and injected into leaves of 4-week-old *Nicotiana benthamiana* plants. After 48 h incubation under dark conditions, mCherry fluorescence was visualized using a confocal laser scanning microscope.

## Figures and Tables

**Table 1:** The biophysical information of MsRCI2 proteins.

Name	Number of Amino Acids	Theoretical pI	Molecular Weight	Instability Index	Stability	Aliphatic Index	GRAVY	Subcellular Localization
MsRCI2As	54	5.82	5931.38	35.83	Y	164.26	1.602	vacu
MsRCI2Bs	54	6.49	5946.35	40.45	N	151.67	1.526	vacu, plas
MsRCI2Cs	54	4.68	6020.44	23.67	Y	162.41	1.607	vacu
MsRCI2D-1	54	4.00	5921.19	31.66	Y	153.15	1.478	vacu
MsRCI2D-2/3	54	4.00	5903.16	31.66	Y	160.37	1.513	vacu
MsRCI2Es	76	5.59	8697.50	59.49	N	138.55	0.851	plas
MsRCI2Fs	77	5.05	8911.71	70.12	N	127.92	0.758	plas, vacu

Note: GRAVY: grand average of hydrophilicity, vacu: vacuolar membrane, plas: plasma membrane.

## Data Availability

The original contributions presented in the study are included in the article/Supplementary Material.

## Supplementary Materials

The following supporting information can be downloaded at: https://www.mdpi.com/article/10.3390/ijms26094165/s1.

## References

[1] Gong Z., Xiong L., Shi H., Yang S., Herrera-Estrella L.R., Xu G., Chao D., Li J., Wang P., Qin F. (2020). Plant abiotic stress response and nutrient use efficiency. Sci. China Life Sci..

[2] Ma L., Ye J., Yang Y., Lin H., Yue L., Luo J., Long Y., Fu H., Liu X., Zhang Y. (2019). The SOS2-SCaBP8 complex generates and fine-tunes an AtANN4-dependent calcium signature under salt stress. Dev. Cell.

[3] Wang X.S., Ren H.L., Wei Z.W., Wang Y.W., Ren W.B. (2017). Effects of neutral salt and alkali on ion distributions in the roots, shoots, and leaves of two alfalfa (*Medicago sativa*) cultivars with differing degrees of salt tolerance. J. Integr. Agric..

[4] Rahman M.A., Woo J.H., Lee S., Park H.S., Kabir A.H., Raza A., Sabagh A.E., Lee K. (2022). Regulation of Na^+^/H^+^ exchangers, Na^+^/K^+^ transporters, and lignin biosynthesis genes, along with lignin accumulation, sodium extrusion, and antioxidant defense, confers salt tolerance in alfalfa (*Medicago sativa*). Front. Plant Sci..

[5] Lu Q., Ge G., Sa D., Wang Z., Hou M., Jia Y.S. (2021). Effects of salt stress levels on nutritional quality and microorganisms of alfalfa-influenced soil. PeerJ.

[6] Wei T., Li G., Cui Y., Xie J., Gao X., Teng X., Zhao X., Guan F., Liang Z. (2024). Variation characteristics of root traits of different alfalfa (*Medicago sativa*) cultivars under saline-alkaline stress and their relationship with soil environmental factors. Phyton-Int. J. Exp. Bot..

[7] Jalil S.U., Ansari M.I. (2020). Physiological role of Gamma-aminobutyric acid in salt stress tolerance. Salt and Drought Stress Tolerance in Plants: Signaling Networks and Adaptive Mechanisms.

[8] Sachdev S., Ansari S.A., Ansari M.I., Fujita M., Hasanuzzaman M. (2021). Abiotic stress and reactive oxygen species: Generation, signaling, and defense mechanisms. Antioxidants.

[9] Tiwari S., Tiwari S., Singh M., Singh A., Prasad S.M. (2017). Generation mechanisms of reactive oxygen species in the plant cell: An overview. Reactive Oxygen Species in Plants: Boon Or Bane-Revisiting the Role of ROS.

[10] Ding D. (2020). Adaptability of high quality alfalfa. Chin. Livest. Poult. Seed Ind..

[11] Bhattarai S., Biswas D., Fu Y.B., Biligetu B. (2020). Morphological, physiological, and genetic responses to salt stress in alfalfa: A review. Agronomy.

[12] Cai H., Li C., Cong C., Zhan L., He K., Dong L., Xu H. (2022). Effect of overexpression of *MsRCI2A/B/C* genes on drought tolerance of alfalfa (*Medicago sativa* L.). J. Northeast Agric. Univ.-Nat. Sci. Ed..

[13] Rocha P.S.C.F. (2016). Plant abiotic stress-related *RCI2/PMP3*s: Multigenes for multiple roles. Planta.

[14] Navarre C., Goffeau A. (2000). Membrane hyperpolarization and salt sensitivity induced by deletion of PMP_3_, a highly conserved small protein of yeast plasma membrane. EMBO J..

[15] Morsy M.R., Almutairi A.M., Gibbons J., Yun S.J., de los Reyes B.G. (2005). The *OsLti6* genes encoding low-molecular-weight membrane proteins are differentially expressed in rice cultivars with contrasting sensitivity to low temperature. Gene.

[16] Imai R., Koike M., Sutoh K., Kawakami A., Torada A., Oono K. (2005). Molecular characterization of a cold-induced plasma membrane protein gene from wheat. Mol. Genet. Genom..

[17] Goddard N.J., Dunn M.A., Zhang L., White A.J., Jack P.L., Hughes M.A. (1993). Molecular analysis and spatial expression pattern of a low-temperature-specific barley gene, *blt101*. Plant Mol. Biol..

[18] Zhao Y., Tong H., Cai R., Peng X., Li X., Gan D., Zhu S. (2014). Identification and characterization of the *RCI2* gene family in maize (*Zea mays*). J. Genet..

[19] Kim H.S., Lee J.E., Jang H.Y., Kwak K.J., Ahn S.J. (2016). *CsRCI2A* and *CsRCI2E* genes show opposite salt sensitivity reaction due to membrane potential control. Acta Physiol. Plant..

[20] Li C., Song T., Zhan L., Cong C., Xu H., Dong L., Cai H. (2021). Overexpression of *MsRCI2A*, *MsRCI2B*, and *MsRCI2C* in Alfalfa (*Medicago sativa* L.) Provides Different Extents of Enhanced Alkali and Salt Tolerance Due to Functional Specialization of MsRCI2s. Front. Plant Sci..

[21] Zhang D., Zhang Z., Li C., Xing Y., Luo Y., Wang X., Li D., Ma Z., Cai H. (2022). Overexpression of *MsRCI2D* and *MsRCI2E* enhances salt tolerance in alfalfa (*Medicago sativa* L.) by stabilizing antioxidant activity and regulating ion homeostasis. Int. J. Mol. Sci..

[22] Kim Y.O., Lim H.G., Kim H.S., Ahn S.J. (2020). Overexpression of *CsRCI2H* enhances salt tolerance in *Camelina sativa* (L.). Plant Biotechnol. Rep..

[23] Ben Romdhane W., Ben-Saad R., Meynard D., Verdeil J.-L., Azaza J., Zouari N., Fki L., Guiderdoni E., Al-Doss A., Hassairi A. (2017). Ectopic expression of Aeluropus littoralis plasma membrane protein gene *AlTMP1* confers abiotic stress tolerance in transgenic tobacco by improving water status and cation homeostasis. Int. J. Mol. Sci..

[24] Liu B., Feng D., Zhang B., Mu P., Zhang Y., He Y., Qi K., Wang J., Wang H. (2012). Musa paradisica RCI complements AtRCI and confers Na^+^ tolerance and K^+^ sensitivity in *Arabidopsis*. Plant Sci..

[25] Zeng H., Xu L., Singh A., Wang H., Du L., Poovaiah B.W. (2015). Involvement of calmodulin and calmodulin-like proteins in plant responses to abiotic stresses. Front. Plant Sci..

[26] Zhou Y., Ge L., Li G., He P., Yang Y., Liu S. (2020). In silico identification and expression analysis of Rare Cold Inducible 2 (*RCI2*) gene family in cucumber. J. Plant Biochem. Biotechnol..

[27] Capel J., Jarillo J.A., Salinas J., Martinez-Zapater J.M. (1997). Two homologous low-temperature-inducible genes from *Arabidopsis* encode highly hydrophobic proteins. Plant Physiol..

[28] Medina J., Ballesteros M.L., Salinas J. (2007). Phylogenetic and functional analysis of *Arabidopsis RCI2* genes. J. Exp. Bot..

[29] Brunetti S.C., Arseneault M.K.M., Gulick P.J. (2018). Characterization of the *Esi3*/*RCI2*/*PMP*_3_ gene family in the *Triticeae*. BMC Genom..

[30] Zhang D., Shen Z., He P., Wang J., Li D., Meng J., Zhang D., You J., Luo Y., Wang X. (2025). The synergistic roles of MsRCI2B and MsRCI2E in the regulation of ion balance and ROS homeostasis in alfalfa under salt stress. Int. J. Biol. Macromol..

[31] Smith C.A., Melino V.J., Sweetman C., Soole K.L. (2009). Manipulation of alternative oxidase can influence salt tolerance in *Arabidopsis thaliana*. Physiol. Plant..

[32] Oh D.-H., Leidi E., Zhang Q., Hwang S.-M., Li Y., Quintero F.J., Jiang X., D’Urzo M.P., Lee S.Y., Zhao Y. (2009). Loss of halophytism by interference with SOS1 expression. Plant Physiol..

[33] Ardie S.W., Liu S., Takano T. (2010). Expression of the AKT1-type K^+^ channel gene from Puccinellia tenuiflora, *PutAKT1*, enhances salt tolerance in *Arabidopsis*. Plant Cell Rep..

[34] Liu X., Cai S., Wang G., Wang F., Dong F., Mak M., Holford P., Ji J., Salih A., Zhou M. (2017). Halophytic NHXs confer salt tolerance by altering cytosolic and vacuolar K^+^ and Na^+^ in *Arabidopsis* root cell. Plant Growth Regul..

[35] Chin D., Means A.R. (2000). Calmodulin: A prototypical calcium sensor. Trends Cell Biol..

[36] Kushwaha R., Singh A., Chattopadhyay S. (2008). Calmodulin 7 plays an important role as transcriptional regulator in *Arabidopsis* seedling development. Plant Cell..

[37] Chu M., Li J., Zhang J., Shen S., Li C., Gao Y., Zhang S. (2018). AtCaM4 interacts with a sec14-like protein, PATL1, to regulate freezing tolerance in *Arabidopsis* in a CBF-independent manner. J. Exp. Bot..

[38] Rao S.S., El-Habbak M.H., Havens W.M., Singh A., Zheng D., Vaughn L., Haudenshield J.S., Hartman G.L., Korban S.S., Ghabrial S.A. (2014). Overexpression of *GmCaM4* in soybean enhances resistance to pathogens and tolerance to salt stress. Mol. Plant Pathol..

[39] Yu S., Wu J., Sun Y., Zhu H., Sun Q., Zhao P., Huang R., Guo Z. (2022). A calmodulin-like protein (CML10) interacts with cytosolic enzymes GSTU8 and FBA6 to regulate cold tolerance. Plant Physiol..

[40] Poovaiah B.W., Du L., Wang H., Yang T. (2013). Recent advances in calcium/calmodulin-mediated signaling with an emphasis on plant-microbe interactions. Plant Physiol..

[41] Chen J., Xu X., Hu Z., Yang S. (2022). Recent Advances on Salt Stress Sensitivity and Related Calcium Signals in Plants. Plant Res..

[42] Sanders D., Pelloux J., Brownlee C., Harper J.F. (2002). Calcium at the crossroads of signaling. Plant Cell.

[43] Li Y., Zeng H., Xu F., Yan F., Xu W. (2022). H^+^-ATPases in plant growth and stress responses. Annu. Rev. Plant Biol..

[44] Tang H., Krishnakumar V., Bidwell S., Rosen B., Chan A., Zhou S., Gentzbittel L., Childs K.L., Yandell M., Gundlach H. (2014). An improved genome release (version Mt4. 0) for the model legume *Medicago truncatula*. BMC Genom..

[45] Le S.Q., Gascuel O. (2008). An improved general amino acid replacement matrix. Mol. Biol. Evol..

[46] Kumar S., Stecher G., Li M., Knyaz C., Tamura K. (2018). MEGA X: Molecular evolutionary genetics analysis across computing platforms. Mol. Biol. Evol..

[47] Letunic I., Bork P. (2016). Interactive tree of life (iTOL) v3: An online tool for the display and annotation of phylogenetic and other trees. Nucleic Acids Res..

[48] Chen C.J., Chen H., Zhang Y., Thomas H.R., Frank M.H., He Y.H., Xia R. (2020). TBtools: An integrative toolkit developed for interactive analyses of big biological data. Mol. Plant.

