# Supplementary material for: Genome-Wide Analysis of the MsRCI2 Gene Family in Medicago sativa and Functional Characterization of MsRCI2B in Salt Tolerance
Source: Int J Mol Sci. 2025 Apr 27;26(9):4165. doi: 10.3390/ijms26094165 (PMC12072170; doi:10.3390/ijms26094165)
Supplement: Supplementary file 1 [file ijms-26-04165-s001.zip › ijms-3552994-supplementary.pdf]

Table. S1 Gene IDs and proposed gene names of RCI2 in alfalfa

gene IDs	proposed gene names
Msa0483000	*MsRCI2F-1*
Msa0483010	*MsRCI2E-1*
Msa0882680	*MsRCI2D-1*
Msa0909990	*MsRCI2D-2*
Msa0947750	*MsRCI2D-3*
Msa1040250	*MsRCI2B-1*
Msa1040260	*MsRCI2A-1*
Msa1040270	*MsRCI2C-1*
Msa1083030	*MsRCI2A-2*
Msa1083040	*MsRCI2C-2*
Msa1083270	*MsRCI2B-2*
Msa1128000	*MsRCI2C-3*
Msa1128010	*MsRCI2A-3*
Msa1128020	*MsRCI2B-3*
Msa1131420	*MsRCI2E-2*
Msa1131430	*MsRCI2F-2*
Msa1264480	*MsRCI2F-3*
Msa1264490	*MsRCI2E-3*
Msa1351830	*MsRCI2C-4*
Msa1351840	*MsRCI2A-4*
Msa1450970	*MsRCI2F-4*
Msa1450980	*MsRCI2E-4*

Supplementary Table. S2 Ion transporter-like proteins that interact with MsRCI2B

Name	Function	CDS (bp)	Length (aa)	MW (kDa)b	PI	TMH	Instability Index	Subcellular localization
MTR_1g108765	ATP synthase	1680	559	59.90	5.80	0	42.35	Mitochondrion
MTR_3g106820	ATP synthase	630	209	23.05	5.75	0	35.37	Chloroplast
MTR_4g103330	ATP synthase	750	249	27.18	9.10	0	35.77	Chloroplast
MTR_4g072050	vacuolar H+-pumping ATPase	495	164	16.55	8.62	4	32.82	Vacuole
MTR_5g021690	vacuolar H+-pumping ATPase	495	164	16.54	8.62	4	28.83	Vacuole
MTR_8g076150	vacuolar H+-pumping ATPase	495	164	16.52	8.62	4	31.65	Vacuole
MTR_2g097580	ZIP metal ion transporter family protein	1011	336	37.22	5.99	9	38.64	Plasma Membrane
MTR_3g081580	ZIP zinc/iron transport family protein	1077	358	38.46	6.38	7	31.71	Plasma Membrane
MTR_1g087040	BZIP transcription factor	453	150	16.99	5.94	0	49.41	Nucleus

MTR_1g112270	C2H2 zinc finger protein	1749	582	61.61	9.35	0	47.42	Nucleus
MTR_3g086630	C3HC4-type RING zinc finger protein	456	151	17.13	5.90	1	72.49	Chloroplast
MTR_4g108830	zinc finger protein	660	219	23.47	5.67	1	38.11	Endoplasmic reticulum
MTR_4g021730	metallothionein	228	75	7.53	4.49	0	74.12	Nucleus
MTR_7g061018	metallothionein	237	78	7.80	4.77	0	38.62	Chloroplast
MTR_3g112400	thioredoxin	258	85	9.54	4.68	0	47.95	Cytosol
MTR_7g009070	thioredoxin H2-like protein	405	134	14.84	5.60	0	39.51	Chloroplast
MTR_2g006290	2Fe-2S ferredoxin superfamily protein	453	150	15.91	4.56	0	53.57	Chloroplast
MTR_1g102680	aluminum induced protein	753	250	26.94	5.40	0	33.97	Chloroplast
MTR_2g022520	aluminum induced protein	711	236	25.43	5.43	0	44.38	Cytosol
MTR_7g101880	membrane magnesium transporter	321	106	11.96	6.49	2	21.71	Vacuole
MTR_7g083790	phosphate carrier protein	1122	373	39.53	9.33	0	41.55	Vacuole
MTR_0041	EIN2-like	1629	542	142.29	5.59	11	43.99	Plasma

s0030	protein, nramp transporter							Membrane

Supplementary Table. S3 Transmembrane transporter-like proteins that interact with MsRCI2B

Name	Function	CDS (bp)	Length (aa)	MW (kDa)b	PI	TMH	Instability Index	Subcellular localization
MTR_2g048720	Inositol transporter 4	1713	570	62.30	8.83	12	38.09	Plasma Membrane
MTR_2g078960	Mate efflux family protein	1446	481	52.99	7.11	12	32.59	Plasma Membrane
MTR_2g096900	Outer envelope pore protein	444	147	15.34	8.89	0	8.20	Chloroplast
MTR_3g115930	transmembrane protein	510	169	17.92	9.67	1	46.45	Chloroplast
MTR_3g073300	transmembrane protein 50A	408	135	14.85	4.38	4	34.58	Vacuole
MTR_3g064040	transmembrane protein	576	191	20.51	7.82	2	49.60	Chloroplast
MTR_4g133770	transmembrane protein	576	191	21.06	9.12	3	32.21	Plasma Membrane
MTR_7g080280	transmembrane protein	513	170	18.68	4.84	1	32.58	Extracellular
MTR_4g133968	oligopeptide transporter OPT family protein	2226	741	83.41	9.25	16	35.64	Plasma Membrane
MTR_4g109720	ABC transporter B family protein	1902	633	68.40	8.18	4	33.88	Plasma Membrane

MTR_8g096320	bidirectional sugar transporter	603	255	22.42	9.80	5	40.15	Plasma Membrane

Supplementary Table. S4 Photosynthesis-related proteins that interact with MsRCI2B

Name	Function	CDS (bp)	Length (aa)	MW (kDa)[b]	PI	TMH	Instability Index	Subcellular localization
MTR_3g068030	ribulose bisphosphate carboxylase/oxygenase activase	1269	422	46.59	6.39	0	30.50	Peroxisome
MTR_4g021210	ribulose bisphosphate carboxylase/oxygenase activase	1431	476	52.17	5.42	0	32.07	Chloroplast
MTR_6g018300	ribulose bisphosphate carboxylase small chain	534	177	19.78	8.72	0	29.30	Chloroplast
MTR_6g018310	ribulose bisphosphate carboxylase small chain	543	180	20.04	8.72	0	32.51	Chloroplast
MTR_3g088040	light-harvesting complex I chlorophyll A/B-binding protein	813	270	28.88	6.40	3	27.60	Plasma Membrane
MTR_3g0703	light-harvesting	837	278	30.67	5.75	3	34.26	Chloroplast

40	complex I chlorophyll A/B-binding protein							
MTR_4g094605	light-harvesting complex I chlorophyll A/B-binding protein	801	266	28.30	5.47	0	23.96	Chloroplast
MTR_8g023790	light-harvesting complex I chlorophyll A/B-binding protein	807	268	28.84	6.42	0	37.52	Chloroplast
MTR_2g064650	photosystem II 10 kDa proteinPsbR protein	384	127	13.56	9.81	0	13.59	Cytosol
MTR_3g086230	photosystem II core complex family psbY protein	594	197	20.50	9.39	4	37.36	Chloroplast
MTR_7g079900	photosystem I reaction center subunit XI	645	214	22.92	9.45	2	39.61	Chloroplast
MTR_8g005175	oxygen-evolving enhancer protein	801	266	28.74	9.51	0	36.75	Chloroplast
MTR_8g078870	photosystem II oxygen-evolving	990	329	35.00	6.25	0	32.20	Chloroplast

	enhancer protein							
MTR_1g070495	photosystem II reaction center	414	137	14.21	5.00	2	63.81	Chloroplast
MTR_4g052420	photosystem I subunit O	417	139	15.24	9.78	2	47.08	Chloroplast
MTR_3g114850	plastocyanin	504	167	17.07	4.99	0	23.89	Chloroplast
MTR_3g462990	thylakoid soluble phosphoprotein TSP9 protein	288	95	10.33	9.85	0	31.28	Mitochondrion
MTR_7g118290	thylakoid membrane phosphoprotein 14 kDa protein	477	158	16.58	5.20	1	47.54	Chloroplast
MTR_4g125600	ultraviolet-B-repressible protein	354	117	11.79	10.01	1	23.82	Chloroplast

Supplementary Table. S5 Enzymes that interact with MsRCI2B

Name	Function	CDS (bp)	MW (kDa)b	PI	TMH	Instability Index	Subcellular localization
MTR_2g012420	NADPH-dependent quinone oxidoreductase	765	27.09	7.60	0	40.91	Cytosol
MTR_4g061140	L-ascorbate peroxidase	741	26.71	5.64	0	33.00	Cytosol
MTR_4g073410	L-ascorbate peroxidase	864	31.73	6.26	1	45.28	Endoplasmic reticulum
MTR_3g088160	thylakoid-bound ascorbate peroxidase	1311	47.47	9.02	1	38.98	Chloroplast
MTR_5g022300	leaf ferredoxin-NADP reductase	762	27.62	8.76	0	29.77	Chloroplast
MTR_3g111190	glutamyl-tRNA reductase family protein	1308	48.40	6.59	0	35.81	Chloroplast
MTR_8g076290	cytochrome P450 family monooxygenase	1533	57.48	6.11	1	38.07	Chloroplast
MTR_7g099720	oligosaccaryltransferase	216	8.18	6.90	1	43.31	Chloroplast
MTR_2g041620	acyl-CoA N-acyltransferase (NAT) superfamily protein	43	32.11	9.43	0	50.48	Nucleus
MTR_3g075440	LRR receptor-like kinase family protein	1887	69.99	9.21	1	35.17	Mitochondrion
MTR_3g062500	LRR receptor-like kinase	1953	70.83	8.40	2	45.70	Plasma Membrane

MTR_8g070880	LRR receptor-like kinase	1953	106.43	5.79	1	39.24	Plasma Membrane
MTR_3g047890	receptor-like kinase plant	1518	56.76	7.30	1	33.99	Chloroplast
MTR_8g083090	S-adenosyl-L-homocysteine hydrolase	1458	53.33	5.62	0	33.89	Cytosol
MTR_7g112640	ATP-dependent Clp protease proteolytic protein	969	35.60	9.13	0	58.34	Chloroplast
MTR_5g026840	ATP-dependent Clp protease proteolytic protein	894	32.51	5.09	0	46.02	Mitochondrion
MTR_3g034670	alpha/beta fold hydrolase	1560	57.99	7.53	0	43.80	Chloroplast
MTR_8g087820	cyclic phosphodiesterase-like protein	555	20.64	5.95	0	36.60	Nucleus
MTR_4g086510	CXE carboxylesterase	966	35.65	5.62	0	29.68	Cytosol
MTR_2g039120	beta-like galactosidase	2004	75.78	8.72	1	37.22	Chloroplast
MTR_5g025830	beta-galactosidase-like protein	2496	93.12	8.35	0	23.22	Chloroplast
MTR_1g048970	pterin-4-alpha-carbinolamine dehydratase	639	23.62	8.47	0	43.03	Chloroplast
MTR_1g023210	papain family cysteine protease	1095	39.94	6.55	0	33.22	Vacuole
MTR_7g08	phosphoglycolate	795	29.67	8.71	0	35.93	Chloroplast

0530	phosphatase-like protein						
MTR_6g011810	trypsin-like serine protease	1377	48.66	6.16	0	37.66	Chloroplast
MTR_6g027620	valyl-tRNA synthetase/valine-tRNA ligase	2943	111.73	6.12	0	42.93	Chloroplast
MTR_5g076620	squalene/phytoene synthase	1176	43.89	8.33	0	50.16	Chloroplast
MTR_2g044070	sucrose synthase	2415	92.30	5.91	0	37.05	Cytosol
MTR_5g022730	SCF ubiquitin ligase, SKP1 component	468	17.59	4.55	0	48.60	Cytosol
MTR_4g075290	peptidyl-prolyl cis-trans isomerase	519	18.19	8.36	0	9.04	Cytosol
MTR_5g032440	threonine endopeptidase	549	20.14	5.42	2	33.32	Chloroplast

Supplementary Table. S6 Primer sequences used in the experiments

Primer name	Primer sequence (5’-3’)
RCI2B	S:ATGGGCACAGCTACATGCATC
	AS:TCACTTGGTAATAGCATAGATAGCATAGA
pBT3--N-RCI2B	S:AGGCCTTTAATTAAGGCCGCCATGGGCACAGCTACATGCATC
	AS:ATTCCTGCAGATGGCCGATCACTTGGTAATAGCATAGATAGCATAGA
LUC-V-ATPase	S:AGTGGTCTCTGTCCAGTCCTATGGCTCCATTCAGCGGCGATG
	AS:GGTCTCAGCAGACCACAAGTCTCGGCTCTAGACTGGCCGGCA
ATP synthase	S:TCAACGAGAAACAGGAAGAACCTT
	AS:ATGGCCTCAATGATCATGGCT
V-ATPase	S:ATGGCTCCATTCAGCGGCGAT
	AS:TTACTCGGCTCTAGACTGGCCGGC
Sugar transporter	S:ATGCTTTGGATCTACTATGCATTTGTC
	AS:TCAGACTTTGCTACCGGCACC
Clp protease	S:ATGGATTTCCTCACAACTTCT
proteolytic protein	AS:CTAGTATATCTCATCATCTGGGAT
Qpcr-V-ATPase	S:TCATCACACCCATGGAAGCC
	AS:GCGATGAAACTGCACCCTTC
Qpcr-CaM	S:CTGTGATGAGGTCGCTAGGC
	AS:CGGGCCATCAGGTTAAGGAA

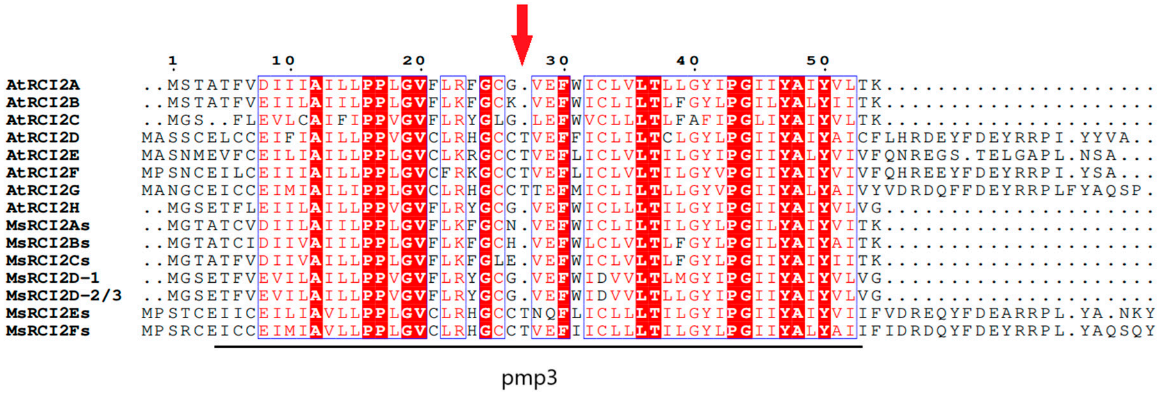

Figure. S1 Sequence comparison among MsRCI2s and other AtRCI2s proteins. The alignment was generated in MEGA11 software.

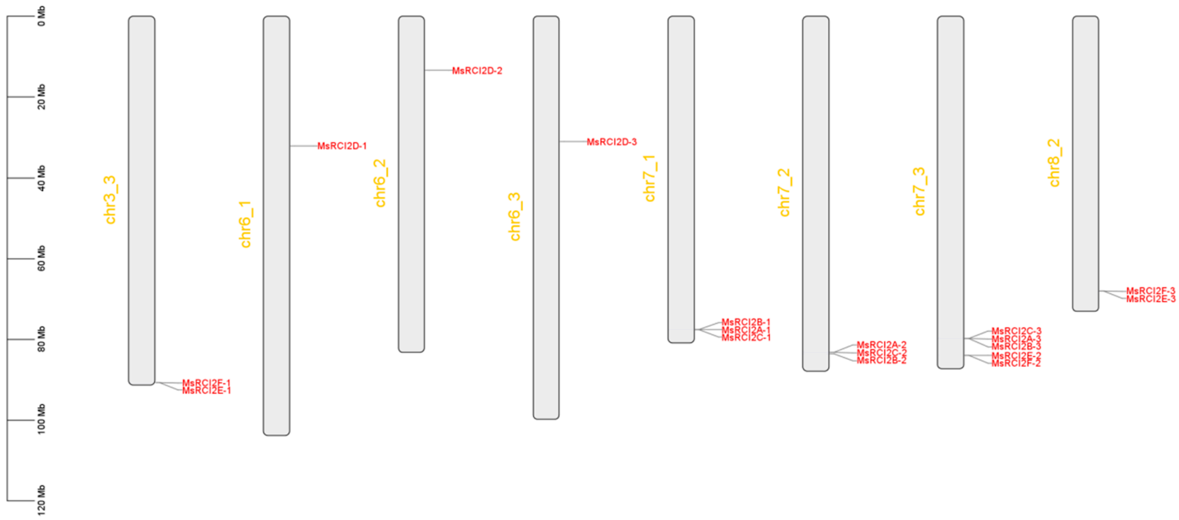

Figure. S2 Chromosomal localization of MsRCI2s.

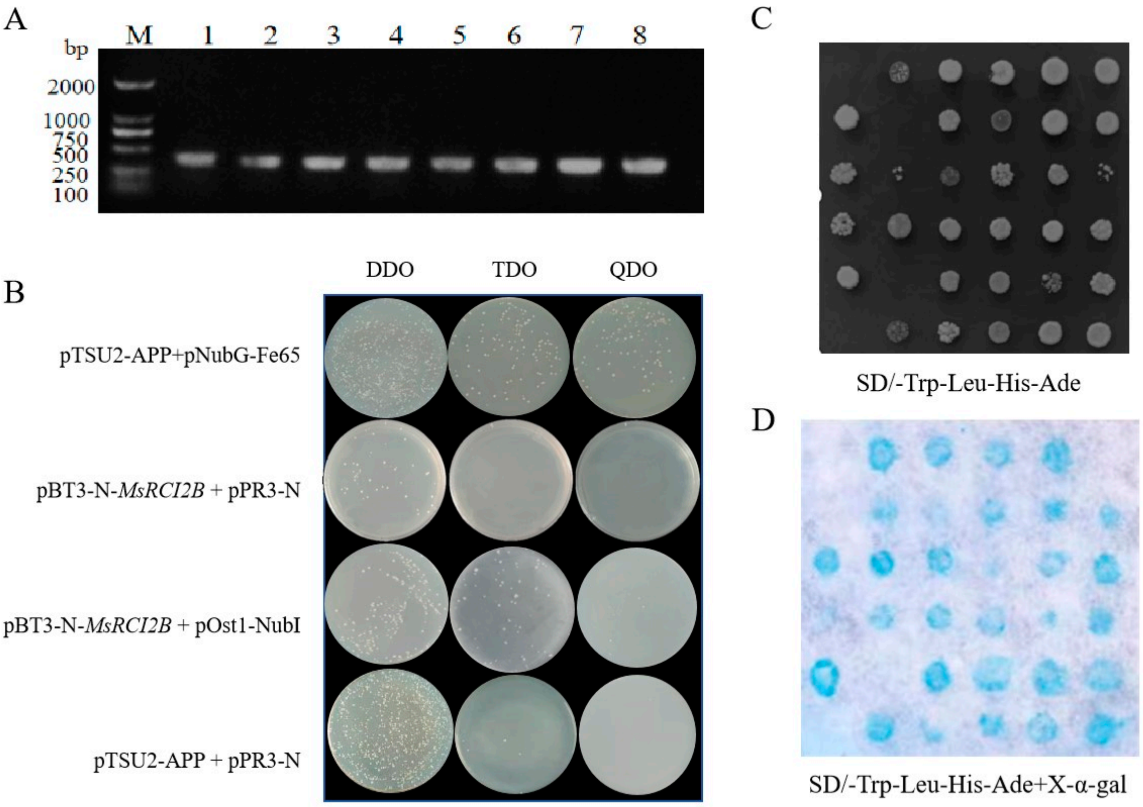

Figure. S3 Construction and screening of a membrane yeast two-hybrid library in *Medicago sativa* L. (A) pBT3-N-MsRCI2B vector construction, M：Marker；1-8：Connection products of pBT3-N-*MsRCI2B;* (B) Self-activation of MsRCI2B protein, DDO is SD/-Trp/-Leu/-His medium, TDO is SD/-Trp/-Leu/-His medium, QDO is SD/-Trp/-Leu/-His/-Ade medium, (+) positive control pTSU2-APP + pNubG-Fe65; (−) negative control pTSU2-APP + pPR3-N (C) Screening of MsRCI2B interacting proteins using the pPR3-N membrane yeast two-hybrid library The selected candidate interacting proteins were applied to a quadruple-deficient medium to assess their growth . (D) the growth of SD/-Trp/-Leu/-His/-Ade with dropping x- α -gal. X-gal was then added to the medium.

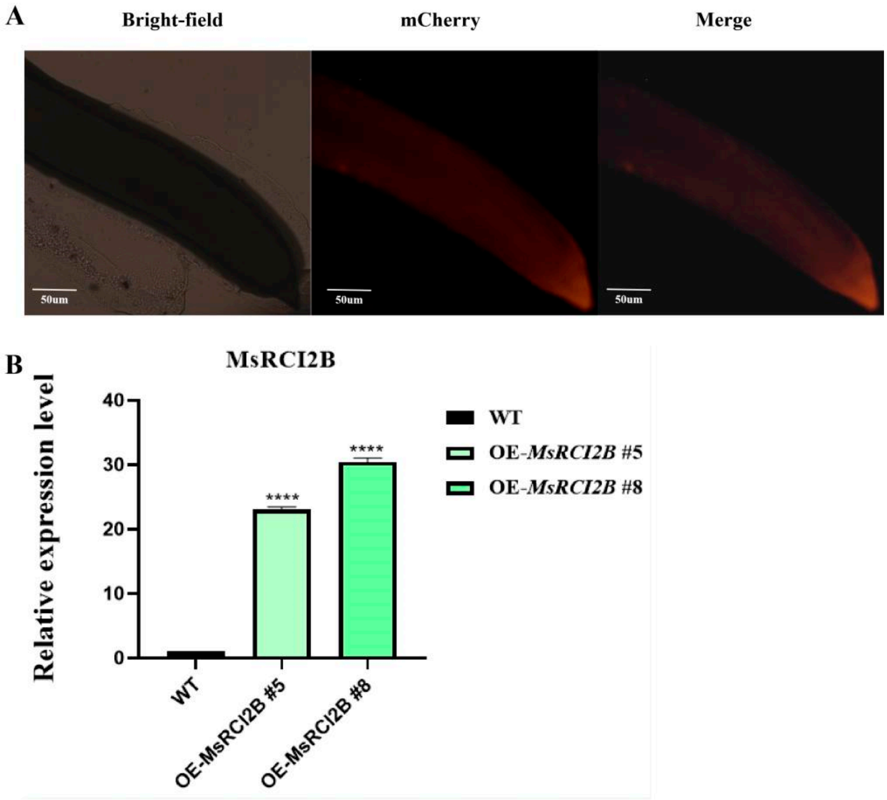

Figure. S4 Molecular characterization of transgenic alfalfa overexpressing MsRCI2B. (A) Detection of mCherry fluorescence in roots of *MsRCI2B*-overexpressing (OE) transgenic lines. Representative confocal microscopy images show mCherry fluorescence signals (red) localized in root tissues of transgenic plants (n = 10). Wild-type (WT) roots exhibited no detectable fluorescence. Scale bar: 50 μ m. White arrows indicate mCherry signal hotspots. (B) Quantitative analysis of *MsRCI2B* gene in transgenic lines. Fluorescence intensity was normalized to WT controls (set as 1.0) and expressed as mean ± SD (n = 3 biological replicates). Significant differences between transgenic lines and WT were determined by turkey test (**** $p < 0.0001$).
